# Modelling the spatial and seasonal distribution of suitable habitats of schistosomiasis intermediate host snails using Maxent in Ndumo area, KwaZulu-Natal Province, South Africa

**DOI:** 10.1186/s13071-016-1834-5

**Published:** 2016-11-04

**Authors:** Tawanda Manyangadze, Moses John Chimbari, Michael Gebreslasie, Pietro Ceccato, Samson Mukaratirwa

**Affiliations:** 1 Department of Public Health Medicine University of KwaZulu-Natal, School of Nursing and Public Health, Durban, South Africa; 2 School of Agriculture, Earth and Environmental Sciences, University of KwaZulu-Natal, Westville, Durban, South Africa; 3 The International Research Institute for Climate and Society, The Earth Institute, Columbia University, Lamont Campus, 61 Route 9 W, Monell Building, Palisades, NY 10964 USA; 4 School of Life Sciences, University of KwaZulu-Natal, Durban, South Africa

**Keywords:** Maxent, Predictive modelling, Snail-borne disease modelling, Schistosomiasis

## Abstract

**Background:**

Schistosomiasis is a snail-borne disease endemic in sub-Saharan Africa transmitted by freshwater snails. The distribution of schistosomiasis coincides with that of the intermediate hosts as determined by climatic and environmental factors. The aim of this paper was to model the spatial and seasonal distribution of suitable habitats for *Bulinus globosus* and *Biomphalaria pfeifferi* snail species (intermediate hosts for *Schistosoma haematobium* and *Schistosoma mansoni*, respectively) in the Ndumo area of uMkhanyakude district, South Africa.

**Methods:**

Maximum Entropy (Maxent) modelling technique was used to predict the distribution of suitable habitats for *B. globosus* and *B. pfeifferi* using presence-only datasets with ≥ 5 and ≤ 12 sampling points in different seasons. Precipitation, maximum and minimum temperatures, Normalised Difference Vegetation Index (NDVI), Normalised Difference Water Index (NDWI), pH, slope and Enhanced Vegetation Index (EVI) were the background variables in the Maxent models. The models were validated using the area under the curve (AUC) and omission rate.

**Results:**

The predicted suitable habitats for intermediate snail hosts varied with seasons. The AUC for models in all seasons ranged from 0.71 to 1 and the prediction rates were between 0.8 and 0.9. Although *B. globosus* was found at more localities in the Ndumo area, there was also evidence of cohabiting with *B. pfiefferi* at some of the locations. NDWI had significant contribution to the models in all seasons.

**Conclusion:**

The Maxent model is robust in snail habitat suitability modelling even with small dataset of presence-only sampling sites. Application of the methods and design used in this study may be useful in developing a control and management programme for schistosomiasis in the Ndumo area.

## Background

Schistosomiasis is a snail-borne disease prevalent in humans [1]. The disease ranks second to malaria in terms of the negative socio-economic effects it has in endemic communities [2–4] mostly in the rural parts of sub-Saharan Africa [5]. The spatial and temporal distribution of intermediate snail hosts that transmit the disease determines the distribution of the disease in endemic areas. People living in rural or semi-rural communities are in constant contact with schistosome-infested water [6]. In South Africa, particularly in KwaZulu-Natal Province both urinary (*Schistosoma haematobium*) and intestinal (*S. mansoni*) schistosomiasis are endemic indicating the presence of the intermediate snail hosts [7–9]. The prevalence of urinary schistosomiasis has been reported to be high (68–80 %) in uMkhanyakude district [10, 11] including Ndumo area. However, spatial and temporal modelling of the snail habitats mainly at a micro-geographical scale to get insights of schistosomiasis transmission dynamics has not been done.

It is essential that the distribution of schistosome intermediate hosts be known for effective design, implementation and evaluation of schistosomiasis control programs [12]. The population dynamics of the intermediate snail hosts and parasite transmission patterns *in situ* are still to be adequately studied [4]. Even with inexpensive and effective anthelminthic medication (Praziquantel) the knowledge of the local dynamics of snail populations is still important for timing of mass drug administration, the global strategy endorsed by the World Health Organization [13]. This will help to follow the periods when re-infection is very low [14–17] so as to reduce the chances of reinfection. The sustainability of this control strategy has been challenged, as there is rapid re-infection after deworming [3, 18]. There has been a shift from morbidity control to transmission control and local elimination [19]. Hence, there is a stronger focus on intermediate snail hosts and transmission sites, along with primary prevention tailored to specific socio-ecological systems [16, 17, 20]. Understanding the dynamics of transmission of schistosomiasis could help to identify hot spots where transmission may be intense and build towards effective local intervention programmes.

Specific habitat requirements of intermediate snail hosts are governed by environmental factors [21]. The intermediate snail hosts need an aquatic environment and thrive even in small water bodies (SWBs), such as ponds, ditches and other humid areas consisting of open water, aquatic vegetation and/or inundated grass [22]. Although snails may reproduce through selfing and aestivate during the dry season [23] triggered by the drying of water pools, live snails are limited to locations with standing water or with enough moisture for survival [24].

Habitats of intermediate snail hosts can be mapped by extensive ground surveys requiring considerable amounts of time, manpower and money. Thus the use of remotely sensed imagery is a useful alternative for habitat detection as it reduces costs, time and manpower [22]. The use of satellite remote sensing data and techniques for risk profiling of environment-related diseases, including schistosomiasis, has increased considerably over the past 30 years [25, 26]. Remote sensing data have been mainly used to relate schistosomiasis prevalence at the school level to remote sensing measurements such as Normalised Difference Vegetation Index (NDVI) and Normalised Difference Water Index (NDWI), to model and spatially predict the risk of infection [15, 25–29]. However, the aim of the application of remote sensing data is to characterize the environmental conditions of potential disease transmission sites, which are in many cases spatially disjunct from the school location where epidemiological surveys are usually being conducted [29].

Modelling habitats of intermediate snail hosts using the Maximum Entropy (Maxent) modelling technique could substantially improve our understanding of the temporal and spatial distribution of current risk of schistosomiasis and create novel possibilities for improved schistosomiasis control and management [22, 25, 30–32]. Maxent is a recently developed ecological modelling method capable of achieving high predictive performance [33] using the presence-only data [33–35] in contrast to the background environmental conditions. For planning of successful interventions against schistosomiasis and to target populations living in high risk areas, it is of great importance to determine the current spatial distribution of infection at a reasonably fine scale, including the distribution of parasites and host species [36]. Detailed maps of possible distribution of habitats of intermediate snail hosts provide valuable information for the prediction of infection risk zones but are currently lacking for most parts of the world [22] There is need for micro-geographical studies on the spatial and temporal distribution of these species to guide the control and management of schistosomiasis especially at the community level. Therefore, the purpose of this paper was to model the spatial and seasonal distribution of suitable habitats of intermediate snail hosts of *Schistosoma* spp. based on climatic and non-climatic factors using Maxent in the Ndumo area, uMkhanyakude district in the KwaZulu-Natal province of South Africa. This method has only been used by Stensgaard [37] in modelling schistosomiasis/snails in Africa at the continental level and Pedersen [36] at the national level.

## Methods

### Study area

**Fig. 1:**
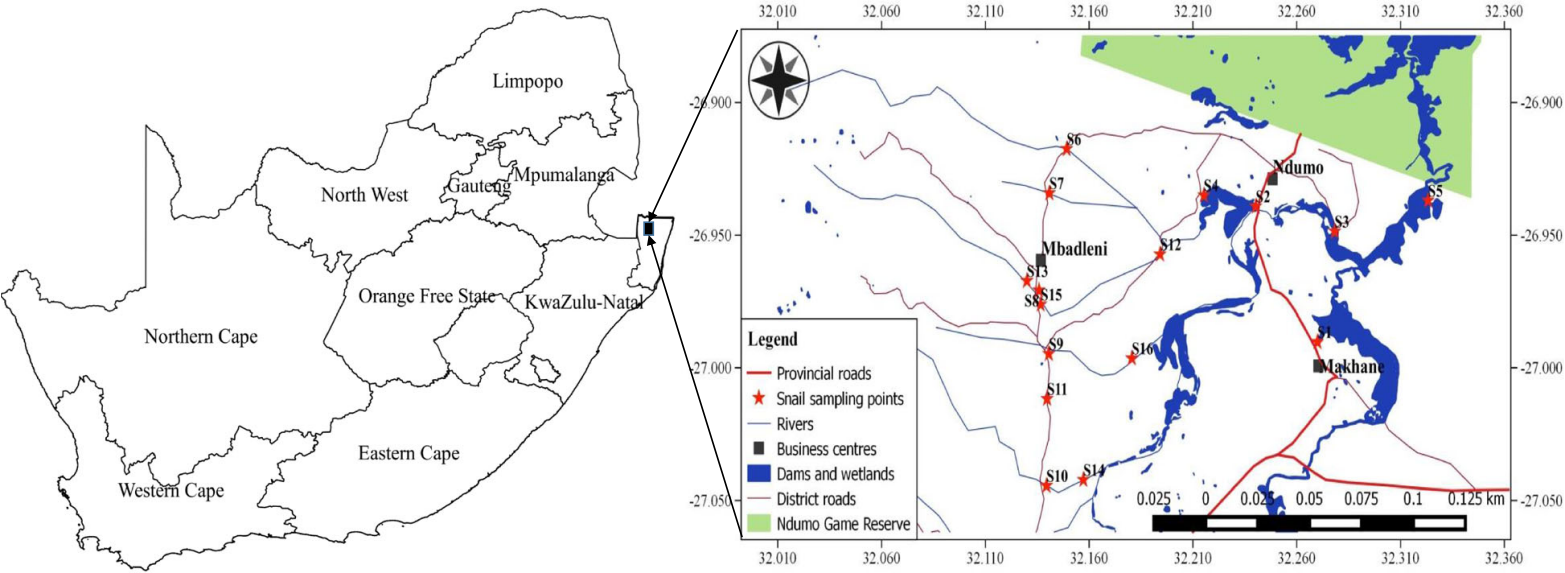
Ndumo area of uMkhanyakude district, KwaZulu-Natal, South Africa

Ndumo area is located in the uMkhanyakude Health District in the KwaZulu-Natal (KZN) province, South Africa (Fig. 1). uMkhanyakude is located in the northenmost eastern part of the KwaZulu-Natal province bordering Mozambique and Swaziland to the north and north-west, respectively. The area is approximately 40 × 30 km mostly characterised by seasonal streams flowing towards the Pongola flood plain. There are two main dams in this area: Nsunduza and Namaneni. The climate of the area ranges from tropical to subtropical [38] experiencing low precipitation averaging at 690 mm per year. The year was divided into four seasons according to temperature and rainfall; rainy (December to February), post-rainy (March to May), cold/dry (June to August) and hot/dry (September to November) based on previous studies in the same region [39–41]. The presence of schistosome intermediate snail hosts (*B. globosus* and *B. pfeifferi*) in uMkhanyakude has been reported by Appleton [9], De Kock et al. [8] and De Kock & Wolmarans [7].

### Snail survey data

The snail sampling points were fairly distributed in the study area (Fig. 1) and were located along rivers, streams and dams targeting suspected transmission sites as advised by Appleton & Miranda [42]. A total of 16 sites were sampled for a period of 1 year (May 2014 to April 2015). The number of sites sampled was considered adequate as the main species modelling technique (Maxent) used in this study has been proven to perform well with a minimum of 10 sampling points [43] and Pearson et al. [44] supported the use of Maxent when sample sizes are very low (<10 but ≥ 5). Maxent also requires presence-only data [33, 34] hence only sites where snails were present in a particular season were used in Maxent seasonal models (Table 1). During the period of sampling *B. globosus* was found in 12 sites and at 7 of those sites both *B. globosus* and *B. pfeifferi* were found. Four sites including S3, S5, S11 and S15 (Fig. 1) had neither *B. globosus* nor *B. pfeifferi*.

**Table 1:** Number of sites used in MAXENT modelling per season in Ndumo area of uMkhanyakude district, South Africa

	Number of sampling sites with intermediate host snails			
Snail species	Cold/dry (June-August)	Hot/dry (September-November)	Rainy (December-February)	Post-rainy (March-May)
*Bulinus globosus*	9	8	2^a^	12
*Biomphalaria pfeifferi*	6	4^a^	0^a^	4^a^

^a^We did not apply the MAXENT model since the numbers of sites were too low

### Climatic and environmental factors

When considering relevant candidate variables to be used in a model, the ecology of the species in question needs to be taken into consideration, hence the climatic and non-climatic predictor variables were selected based on their perceived biological relevance for host snail distributions [36, 37, 45–47]. Specifics and sources of the climatic and environmental variables used in this study are listed in Table 2. We focussed on 2 overall classes of environmental variables that have been shown to influence host snail distribution patterns [48, 49] namely (i) climatic variables (temperature and precipitation) and (ii) natural habitat conditions (water bodies and soil conditions) [37]. The survival and reproduction rates of snails in relation to temperature have been described in a number of studies [47, 48, 50–53]. The diurnal temperature range was chosen to account for the demonstrated importance of fluctuating temperatures, as previously shown for *B. pfeifferi* [54]. Temperature of the warmest and coldest quarter was considered to account for the sensitivity of snails to temperature extremes [53, 55]. Seasonal precipitation was used as a measure of the availability of suitable temporary water bodies that snails are known to inhabit [37]. Soil pH was considered as it has been shown to influence pH in water bodies [36, 56, 57]. A 3 month average (March-May) for maximum temperature (T_max_), minimum temperature (T_min_), Normalised Difference Vegetation Index (NDVI), Normalised Difference Water Index (NDWI), Enhanced Vegetation Index (EVI) were used as they have been used by Pedersen et al. [36]. In this study we considered these factors at 2 levels: annual averages and seasonal averages to model the variation of suitable habitats of the host snails at micro-geographical scale.

**Table 2:** Specifics and sources of the environmental data used for habitat suitability modelling

Variables	Data source	Resolution	Reference
Rainfall	UCSB	5 km	ftp://ftp.chg.ucsb.edu/pub/org/chg/products/CHIRPS-2.0/
T_max_	USGS	1 km	http://modis.gsfc.nasa.gov/data/dataprod/mod11.php
T_min_	USGS	1 km	http://modis.gsfc.nasa.gov/data/dataprod/mod11.php
Slope	NASA	1 km	http://www2.jpl.nasa.gov/srtm
Soil pH	ISRIC-WISE	1 km	http://www.isric.org
NDVI	USGS	250 m	http://modis.gsfc.nasa.gov/data/dataprod/mod13.php
EVI	USGS	250 m	http://modis.gsfc.nasa.gov/data/dataprod/mod13.php

*Abbreviations*: *T*_*max*_ maximum temperature, *T*_*min*_ minimum temperature, *NDVI* Normalised Difference Vegetation Index, *EVI* Enhanced Vegetation Index, *UCSB* University of California Santa Barbara, *USGS* United States Geological Survey, *NASA* National Aeronautics and Space Administration, *ISRIC-WISE* International Soil Reference and Information Centre - World Inventory of Soil Emission Potentials

The Normalised Difference Water Index (NDWI) was calculated based on Moderate Resolution Imaging Spectroradiometer (MODIS) reflectance and temperature amplitude was calculated from MODIS minimum and maximum temperature. Climate Hazards Group InfraRed Precipitation with Station data (CHIRPS) was recorded for 30 years (January 1981 to August 2014) and other datasets from MODIS started between 2000 and 2003 − 2014. We used these datasets as they have higher spatial resolution; most of the datasets were accessed through the International Research Institute for Climate and Society (IRI) data library portal (http://iridl.ldeo.columbia.edu/SOURCES/).

We performed a descriptive statistical analysis (means and standard deviations, SD) of climatic and environmental variables to determine their spatial variation annually and in different seasons. We also removed highly correlated variables (>0.9) [58] as the multicollinearity may violate statistical assumptions and may alter model predictions [59]. NDVI was highly correlated with other variables hence was removed from the models in other seasons.

### Predictive model implementation

*Bulinus globosus* and *Biomphalaria pfeifferi* species distribution models were developed using the Maximum Entropy (Maxent) [33–35] approach using the species distribution models (SDM) toolbox developed by Brown et al. [60] and implemented in ArcGIS 10.2.

Model performance was expressed as the area under (the receiver operator characteristic) curve (AUC) [61] supported by sensitivity and specificity [62]. An AUC value of 0.5 indicates that the model predicts no better than a random model, while AUC values of > 0.75 are considered in the “best” model category [33]. However, comparing models across species using AUC scores is problematic, as AUC is influenced by species’ prevalence [61]. This issue was alleviated by only comparing AUC values among models within species [37]. The spatial jackknifing or geographically structured k-fold validation which test evaluation performance of spatially segregated localities was also used in this study [60, 63]. Hence we considered the omission rate and model feature class complexity instead of the specificity and sensitivity since we used small sample sizes (<25) which could have inflated the performance of the model. The spatial jackknifing script in the SDM toolbox chooses the best model by evaluating each model's omission rates (OR), AUC and model feature class complexity. A jackknife procedure, implemented in Maxent, was used to quantify the explanatory power of each environmental variable. “Maximum training sensitivity plus specificity statistics” output from Maxent as a threshold criterion was used in partitioning of the observations into suitable and unsuitable habitats following the recommendation by Hu & Jiang [64]. We then calculated the area for the suitable habitats and also expressed it as a percentage of the total habitat for each model to quantify the differences in habitats in different seasons and between *B. globosus* and *B. pfeifferi*.

## Results

### Variation of climatic and environmental factors

The distribution of habitats of intermediate snail hosts is influenced by the variation or changes in environmental and climatic factors. Tables 3 and 4 indicate the spatial variability of these factors annually and seasonally, respectively in the Ndumo area of uMkhanyakude district in South Africa.

**Table 3:** Spatial variability of the mean climatic and environmental factors in Ndumo area, uMkhanyakude using annual data (*n* = 580)

Layer	Min	Max	Mean	SD
T_ampl_ (°C)	12.2635	20.0988	17.1146	1.4854
EVI	0.1229	0.4863	0.3339	0.0455
NDVI	0.3412	0.7954	0.5870	0.0672
NDWI	0.0572	0.6366	0.3181	0.1017
pH	0.0000	7.1000	6.2607	0.3466
Rainfall (mm)	63.9332	82.6011	67.7048	3.9479
Slope (degrees)	0.0000	8.7760	1.7606	1.5461
T_max_ (°C)	28.6419	36.0420	33.2673	1.4028
T_min_ (°C)	14.5396	17.2790	16.1348	0.6313

*Abbreviations*: *T*_*ampl*_ temperature amplitude (°C), *EVI* Enhanced Vegetation Index, *NDVI* Normalised Difference Vegetation Index, *NDWI* Normalised Difference Water Index, *SD* standard deviation, *T*_*max*_ maximum temperature, *T*_*min*_ minimum temperature

**Table 4:** Seasonal variability of the mean climatic and environmental factors in Ndumo area, uMkhanyakude, South Africa (*n* = 580)

Variable	Rainy	Post-rainy	Cold/dry	Hot/dry
	Mean ± SD	Mean ± SD	Mean ± SD	Mean ± SD
T_ampl_ (°C)	15.29 ± 1.62	14.50 ± 1.34	17.23 ± 1.36	20.74 ± 1.88
EVI	0.43 ± 0.05	0.37 ± 0.04	0.26 ± 0.05	0.28 ± 0.05
NDVI	0.68 ± 0.06	0.66 ± 0.06	0.51 ± 0.08	0.50 ± 0.07
NDWI	0.46 ± 0.08	0.44 ± 0.10	0.21 ± 0.13	0.18 ± 0.11
Rainfall (mm)	91.19 ± 5.51	41.28 ± 3.73	12.76 ± 1.39	62.64 ± 3.21
T_max_ (°C)	35.53 ± 1.58	31.11 ± 1.11	29.11 ± 1.15	36.72 ± 1.85
T_min_ (°C)	20.15 ± 0.42	16.48 ± 0.87	11.85 ± 0.96	15.98 ± 0.59

*Abbreviations*: *EVI* Enhanced Vegetation Index, *NDVI* Normalised Difference Vegetation Index, *NDWI* Normalised Difference Water Index, *SD* standard deviation, *T*_*ampl*_ temperature amplitude, *T*_*max*_ maximum temperature, *T*_*min*_ minimum temperature

### Spatial distribution of schistosome host snail habitats

**Fig. 2:**
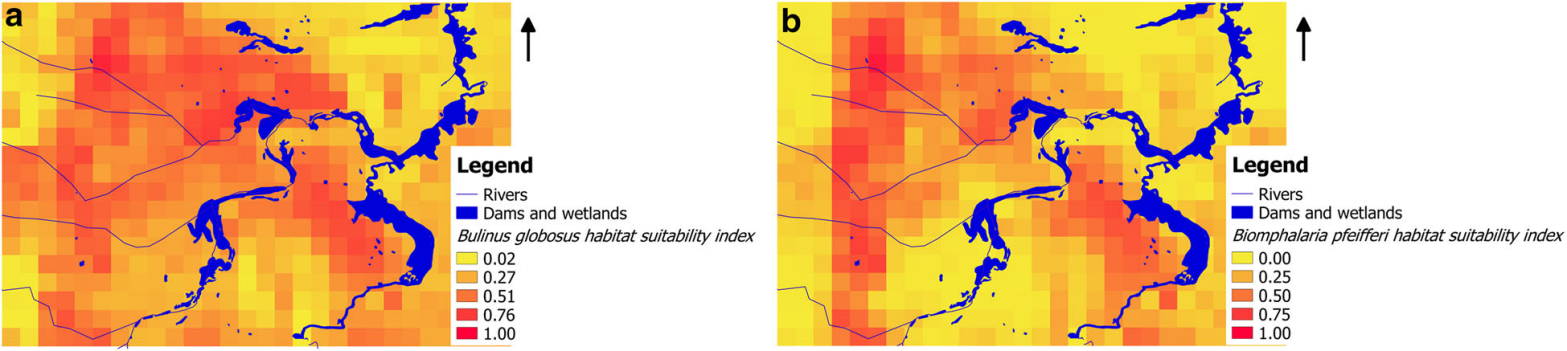
Modelled predictions of habitat suitability of **a** *Bulinus globosus* and **b** *Bimphalaria pfeifferi* in Ndumo area, uMkhanyakude district, South Africa based on annual averages of climatic variables and non-climatic variable (shown in Table 2)

**Fig. 3:**
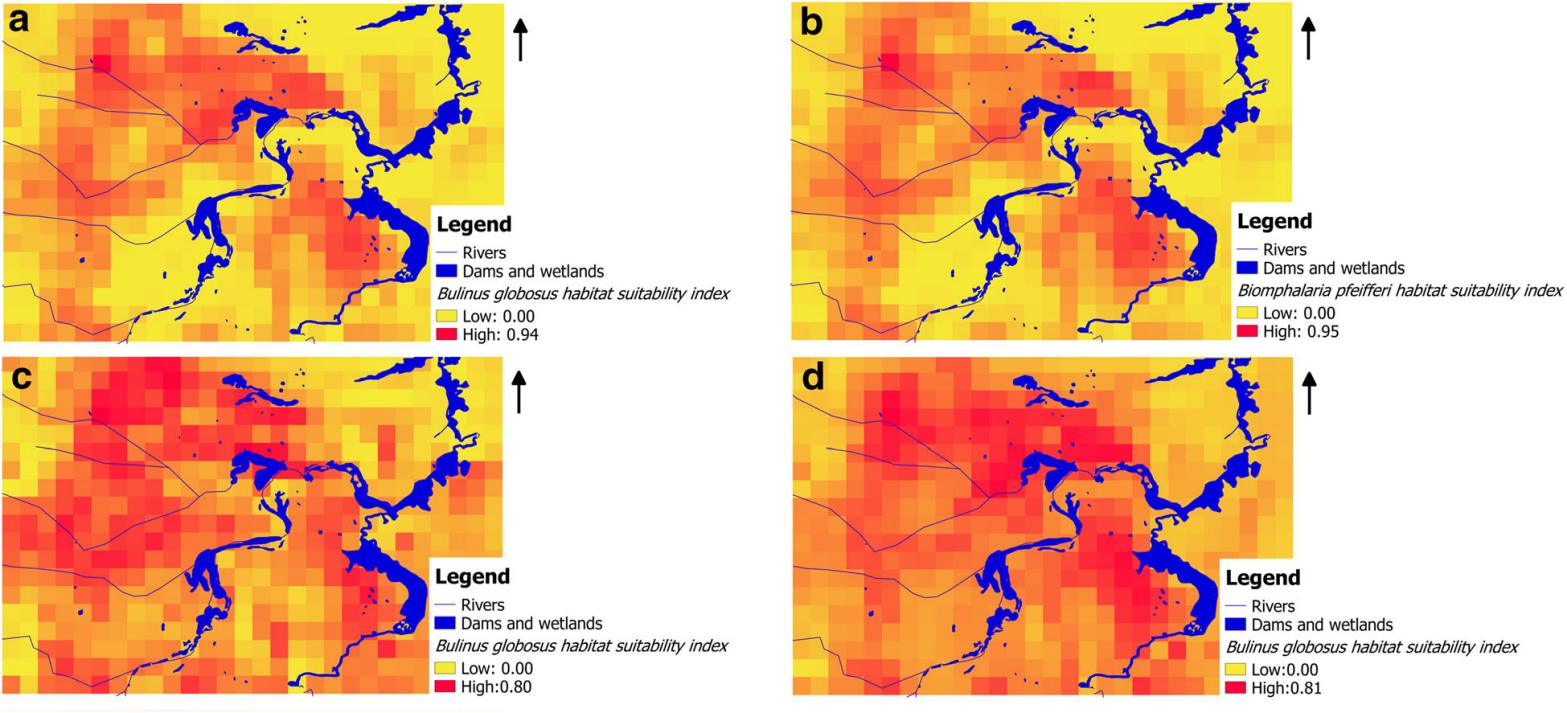
Predicted snail habitat suitability for two snail species in Ndumo area of uMkhanyakude district, South Africa. **a** *Bulinus globosus* in cold/dry season (June to August). **b** *Biomphalaria pfeifferi* in cold/dry season (June to August). **c** *Bulinus globosus* in hot/dry season (September to November). **d** *Bulinus globosus* in post-rainy season (March to May)

The predicted distribution of *B. globosus* and *B. pfeifferi* habitats suitability based on annual averages of climatic variables and non-climatic variables (shown in Table 3) is depicted in Fig. 2. The model predictions indicate that Ndumo area may have more locations with relatively higher probabilities of finding suitable habitats for *B. globosus* compared to *B. pfeifferi*. Figure 3 shows the predicted snail habitats suitable for the two snail species (*B. globosus* and *B. pfeifferi*) in different seasons in the Ndumo area. The possibility of cohabiting of the two species and focality in suitable habitats is also depicted in the two model outputs (Figs. 2 and 3).

**Fig. 4:**
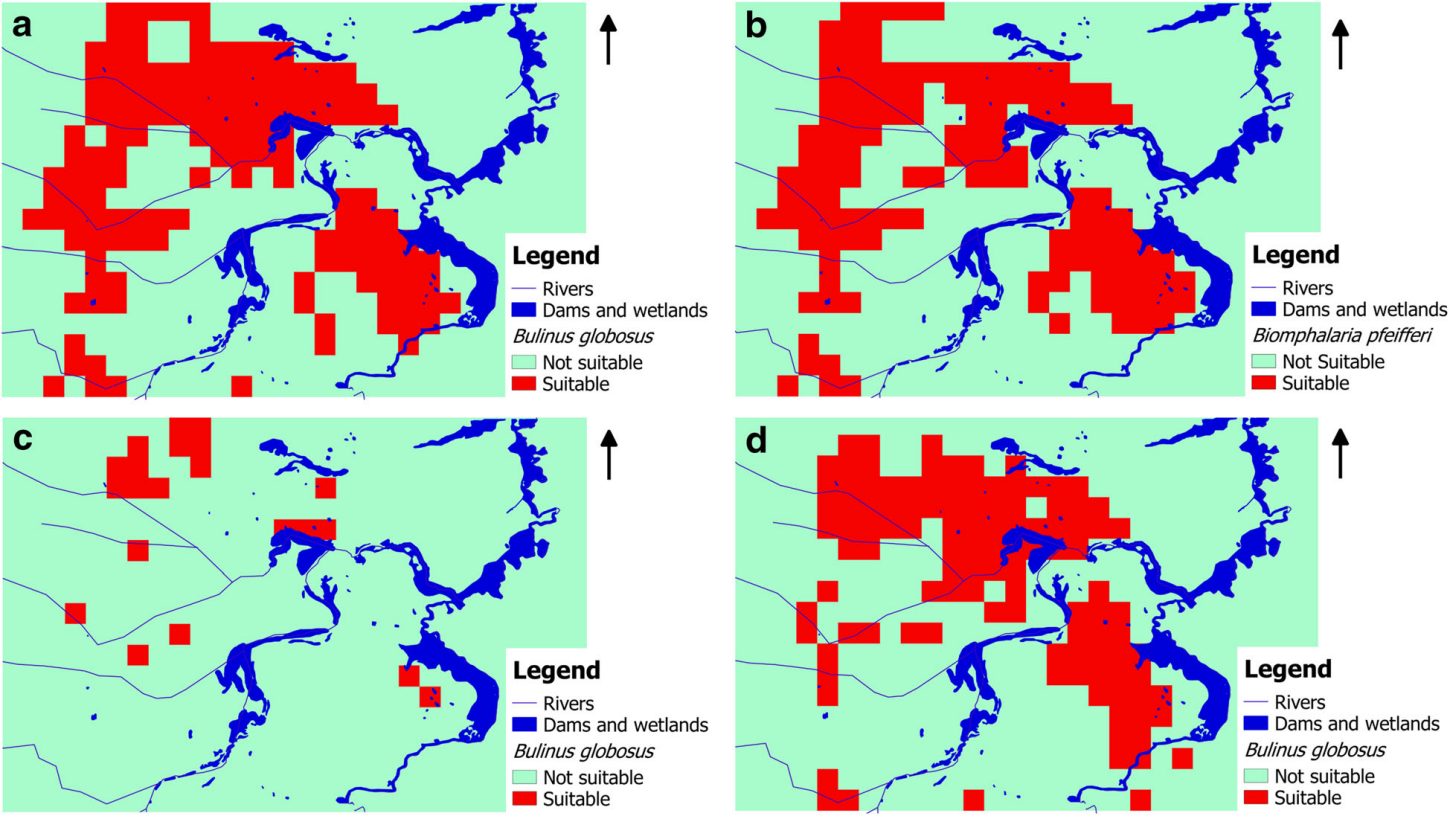
Seasonal suitable and not suitable habitats for two snail species in Ndumo area of uMkhanyakude district, South Africa classified based on maximum training sensitivity plus specificity from Maxent model. **a** *Bulinus globosus* in cold/dry season (June to August). **b** *Biomphalaria pfeifferi* in cold/dry season (June to August). **c** *Bulinus globosus* in hot/dry season (September to November). **d** *Bulinus globosus* in post-rainy season (March to May)

The habitat threshold values used to determine the suitable and unsuitable areas were different for each model (Table 5). Figure 4 shows the variation in suitable areas for the host snails in different seasons classified based on maximum training sensitivity plus specificity from Maxent model.

**Table 5:** Test statistics for two MAXENT models from two snail species in Ndumo area, uMkhanyakude, KwaZulu-Natal, South Africa

	*Bulinus globosus*				*Biomphalaria pfeifferi*	
	Annual	Post-rainy	Cold/dry	Hot/dry	Annual	Cold/dry
AUC	0.71	0.91	1	0.86	0.87	0.8
Weighted prediction^a^	0.80	0.83	0.89	0.86	0.90	0.89
Feature type^b^	4	5	4	4	4	4
Variable contribution (%)						
T_ampl_	24.8	na	0	41.1	4.5	0
EVI	3.9	0	0	5.6	0	0
NDWI	42.3	52	54.3	22.5	53.4	37.7
pH	0	0	0	0	0	0
Rainfall	3.9	27.7	0	3.5	24.6	1.1
Slope	22.1	5	7.1	27.5	4.3	3.1
T_max_	na	15.2	38.6	na	na	58.5
T_min_	3	0	0	0	13.1	0.6
NDVI	na	0.1	na	na	na	na
Habitat threshold value^c^	0.468	0.513	0.328	0.686	0.630	0.356
Suitable habitat area (km^2^)	125.21	87.04	110.71	16.03	62.61	101.54
Percent suitable habitats (%)	29.29	20.36	25.89	3.75	14.64	23.75

^a^The highest weighted prediction indicates the lowest omission rate

^b^Feature type classes: 1, linear; 2, linear and quadratic; 3, hinge; 4, linear, quadratic, and hinge; 5, linear, quadratic, hinge, product, and threshold

^c^Habitat threshold values were based on maximum training sensitivity plus specificity from Maxent model

*Abbreviations*: *AUC* area under the curve, *EVI* Enhanced Vegetation Index, *NDWI* Normalised Difference Water Index, *NDVI* Normalised Difference Vegetation Index, *T*_*max*_ maximum temperature, *T*_*ampl*_ temperature amplitude, *T*_*min*_ minimum temperature, *na* not applicable, the variable was not used due to its high correlation with other variables

The AUC values ranged from 0.71 to 1 for *B. globosus* models and 0.80 to 0.87 for *B. pfeifferi* respectively, indicating good model performance. The weighted prediction for the best performing models was high, ranging from 0.80 to 0.89 for *B. globosus* and 0.89 to 0.90 for *B. pfeifferi* models. The performance of Maxent models in modelling the spatial distribution of *B. globosus* and *B. pfeifferi* and the related factors as well as the variation in the suitable habitats is shown in the Table 5.

NDWI had a consistent high contribution to all of the models in Table 5 regardless of the season. Slope only showed significant contribution on *B. globosus* annual and hot/dry season models. Maximum temperature had a higher contribution to both *B. globosus* and *B. pfeifferi* cold/dry season models. Temperature amplitude had higher significance in *B. globosus* and annual and hot/dry season models. *Bulinus globosus* had more suitable areas in the cold/dry season compared to *B. pfeifferi* (Table 5). However, the two species shared most of the localities. Using annual averages we found that there are more areas suitable for *B. globosus* compared to *B. pfeifferi* (Table 5). *Bulinus globosus* showed the highest percentage of suitable area in the cold/dry season compared to post-rainy and hot/dry seasons, with lowest values in the hot/dry season. This is explained by the tolerance of these snail species (*B. globosus* and *B. pfeifferi*) to the variations in climatic and environmental factors (Tables 3 and 4).

## Discussion

The objective of this study was to model and predict the distribution of suitable habitats for *B. globosus* and *B. pfeifferi* at the micro-geographical scale. Maxent models [33–35] do not predict the actual limits of a species’ range but can identify localities with similar conditions for occurrence [44]. Models presented in our study have indicated a good estimation of the distribution of suitable habitats of schistosome intermediate snail hosts at a micro-geographical scale based on climatic and environmental factors. The results of this study have supported the well-known establishment that Maxent is an efficient tool in modelling species distribution when the datasets are small [43, 44, 65]. In our case we had other seasons with very low numbers of sampling points (<5) and were considered not suitable for obtaining meaningful results [44, 60]. According to the model performance evaluation criteria by Phillips & Dudík, [33] the Maxent model used in this study performed satisfactorily as indicated by the high AUC values which exceeded 0.75 except in 1 case where it was 0.71. However the spatial jackknifing method [60] used in the model evaluation in this study considered omission rate first as the key measure of model performance hence the same model had higher prediction rate (0.8). The Maxent model performance was also appraised by Pedersen et al. [36] and Stensgaard et al. [37] on intermediate snail host habitat suitability modelling. The two species have different foci but overlap at most of the locations in our study area. Similar observations were made by Pedersen et al. [36] based on a national scale study in Zimbabwe. The suitable habitats contract and expand as determined by the environmental and climatic factors as the cold/dry season models showed a wider niche for *B. globosus* compared to post-rainy and hot/dry seasons. However, there are still important conceptual uncertainties in these models which need to be investigated, especially identification of causal relationships between species distribution and predictors [65]. The small sample size and the jacknifing validation method used in this study may inflate the accuracy of the models, hence there is need to assess the performance of this model in a different setting.

We also observed that snail presence probability varied by locations indicating differences in location suitability for the two species, *B. globous* and *B. pfeifferi*. The two snail species showed different levels of sensitivity to different climatic and environmental factors in terms of their suitable habitats. However, NDWI which is an indication of surface water, was the most consistent and significant variable in both species models in all seasons. There are large areas in the eastern parts of our study area where there are water bodies but do not seem to be places where lots of snails are expected. This might be due to factors other than surface water (as detected with NDWI) such as the slope and temperatures which do not favour the presence of host snails. The use of “presence only” data may exclude from further consideration certain habitat types that are deemed falsely unsuitable thereby limiting the creation of models that accurately discriminate between suitable and unsuitable habitats [66]. Our results are slightly different from those of Pedersen et al. [36] who conducted a similar study at the national scale in Zimbabwe which is experiencing almost similar climatic conditions as our study area. Their study showed that *B. globosus* distribution was more influenced by the maximum temperature in the post-rain season (March to May) while *B. pfeifferi* was more influenced by the minimum temperature in that same season. The difference is mainly because Pedersen et al. [36] only considered these variables from 1 season unlike in this current study where we considered different seasons. pH did not show any contribution to the spatial distribution of the host snails. This is contrary to observations by Pedersen et al. [36] where pH showed a significant contribution to the presence of these species. Our study is mainly local and may have lacked significant spatial variation in terms of soil and vegetation types which determine the spatial variation in pH.

Precipitation indirectly affects snails as it indicates the probability of an aquatic environment. However, this variable did not show higher significance levels in the models in our study. Precipitation is related to NDVI, EVI and NDWI but they did not show high correlations. After the rainy season, there are higher chances of finding snails in their preferred habitats since the water bodies are reduced in volume and snail densities are at their peak with many snails having grown to full size [36]. Since suitable habitats mainly for *B. globosus* have shown a seasonal peak (cold/dry season in our study) and is strongly influenced by NDWI which is a measure of surface water, the inclusion of multi-temporal classification of remote sensing images for surface water detection as noted by De Roeck et al. [22] could improve spatial distribution model outputs. The determined relationships may be used to predict possible spatial and temporal changes or variation in snail habitats and snail densities over the past and future projections complementary to Maxent models. It is unfortunate that we did not have enough sampling sites to run Maxent models for all the seasons for both species as some sites became dry during the dry season. In this study the rainy season (December to February) had too few positive data points for further Maxent analysis and it was anticipated that there could be more suitable areas based on increased rainfall which may increase surface water which is critical for snail habitats. However, Anderson [67], noted that it is difficult to measure the spatial relationship between rainfall and snail population dynamics and infection transmission since the effect of rainfall varies depending on the species of snail and the geographical location. Although snails do not thrive without water as they tend to aestivate and may not be detected easily for such studies, too much water may also reduce snail populations [25] especially in fast flowing rivers or streams. However these results give insight into the spatial and temporal dynamics of the suitable habitats for intermediate snail hosts which is critical for control, monitoring and management of the disease.

Although the presence of snails is necessary to determine an area of schistosomiasis transmission the mere presence of snails is not sufficient to define a transmission site. If the snails are not infected and there is no human-water contact, transmission does not occur. Thus, while useful, this model alone may not be adequate for developing a schistosomiasis control, monitoring and management scheme. Notwithstanding this, our model was able to confirm observations made by Moodley et al. [68] and Pitchford [69] based on minimum temperature suitable for schistosomiasis transmission; and by Manyangadze et al. [70] and Saathoff et al. [10] through parasitological surveys, mainly for *S. haematobium*. Thus our model complements the efforts made using different methodologies to understand the dynamics of schistosomiasis in this area. Combining the spatial distribution models of schistosomiasis based on environmental and socio-economic factors [70] and the current model (distribution of potential habitats for schistosome intermediate snail hosts) may help to develop effective schistosomiasis control strategies. This could also be supported by the data on the distribution of infected snails and human-water contact sites.

## Conclusions

This study intended to model the spatial distribution of the suitable habitats for *B. globosus* and *B. pfeifferi* at a micro-geographical scale. The method (Maxent) used is robust in modelling suitable habitats for the host snails even with a small dataset of presence-only sampling sites. The results showed that suitable habitats of the schistosome intermediate snail hosts *B. globosus* and *B. pfiefferi* may vary at the micro-geographical scale. Although *B. globosus* was found at more localities in the Ndumo area, there was also evidence of cohabiting with *B. pfiefferi* at some of the locations. NDWI, which is a proxy for surface water was more significant and consistent in its contribution to the models in all seasons. The methods and design used in this study give informative results which may help in control, monitoring and management of schistosomiasis in the area.

## Abbreviations

AUC: Area under (the receiver operator characteristic) curve
CHIRPS: Climate Hazards Group InfraRed Precipitation with Station data
EVI: Enhanced Vegetation Index
IRI: International Research Institute for Climate and Society
ISRIC-WISE: International Soil Reference and Information Centre - World Inventory of Soil Emission Potentials
MAXENT: Maximum entropy
MODIS: Moderate resolution imaging spectroradiometer
NASA: National aeronautics and space administration
NDVI: Normalised difference vegetation index
NDWI: Normalised difference water index
OR: Omission rate
SD: Standard deviation
SDM: Species distribution model
Tmax: Maximum temperature
Tmin: Minimum temperature
UCSB: University of California Santa Barbara
USGS: United States geological survey
